# Cubitanoids and Cembranoids from the Soft Coral *Sinularia nanolobata*

**DOI:** 10.3390/md14080150

**Published:** 2016-08-09

**Authors:** Chih-Hua Chao, Chia-Yun Wu, Chiung-Yao Huang, Hui-Chun Wang, Chang-Feng Dai, Yang-Chang Wu, Jyh-Horng Sheu

**Affiliations:** 1 School of Pharmacy, China Medical University, Taichung 404, Taiwan; chchao@mail.cmu.edu.tw (C.-H.C.); yachwu@mail.cmu.edu.tw (Y.-C.W.); 2 Chinese Medicine Research and Development Center, China Medical University Hospital, Taichung 404, Taiwan; 3 Department of Marine Biotechnology and Resources, National Sun Yat-sen University, Kaohsiung 804, Taiwan; eleahant111@yahoo.com.tw (C.-Y.W.); huangcy@mail.nsysu.edu.tw (C.-Y.H.); wanghc@kmu.edu.tw (H.-C.W.); 4 Ph.D. Program in Translational Medicine, College of Medicine and Ph.D. Program in Toxicology, College of Pharmacy, Kaohsiung Medical University, Kaohsiung 807, Taiwan; 5 Graduate Institute of Natural Products, Kaohsiung Medical University, Kaohsiung 807, Taiwan; 6 Institute of Oceanography, National Taiwan University, Taipei 112, Taiwan; corallab@ntu.edu.tw; 7 Center for Molecular Medicine, China Medical University Hospital, Taichung 404, Taiwan; 8 Department of Medical Research, China Medical University Hospital, China Medical University, Taichung 404, Taiwan; 9 Frontier Center for Ocean Science and Technology, National Sun Yat-sen University, Kaohsiung 804, Taiwan

**Keywords:** *Sinularia*, cubitane, nanoculones A and B, nanolobols A–C

## Abstract

Two new cubitanoids, nanoculones A and B (**1** and **2**), and three new cembranoids, nanolobols A–C (**3**–**5**), as well as six known compounds, calyculone I (**6**), sinulariuol A (**7**), sinulariols C, D, H, and J (**8**–**11**), were isolated from the soft coral *Sinularia nanolobata*, collected off the coast of the eastern region of Taiwan. Their structures were elucidated on the basis of extensive spectroscopic analysis. Cytotoxicity of compounds **1**–**11** was evaluated. The nitric oxide (NO) inhibitory activity of selected compounds was further measured by assay of lipopolysaccharide (LPS)-stimulated NO production in activated RAW264.7 cells. The results showed that none of **1**–**11** exhibited cytotoxicity against the tested cancer cell lines, whereas compound **8** was found to significantly reduce NO production.

## 1. Introduction

Cubitane-type diterpenoids are a rare group of compounds that are isolated from termites, soft corals, and plants [1]. The first cubitanoid, (+)-cubitene, was discovered from the secretion gland of the termite *Cubitermes umbratus* [2]. Afterward, an array of cubitanoids, namely, calyculones A–I, were found from soft corals of genus *Eunicea* [3,4,5]. In some plants, cubitene was detected by gas chromatography–mass spectrometry (GC/MS) from their volatile oils [6]. In the aforementioned three sources, cubitanoids were usually found in company with cembranoids [1], which represent a large group of compounds in soft coral [7,8,9,10]. The origin of cubitanoids was speculated to be associated with the co-occurring cembranoids based on a photochemical interconversion [4].

**Figure 1:**
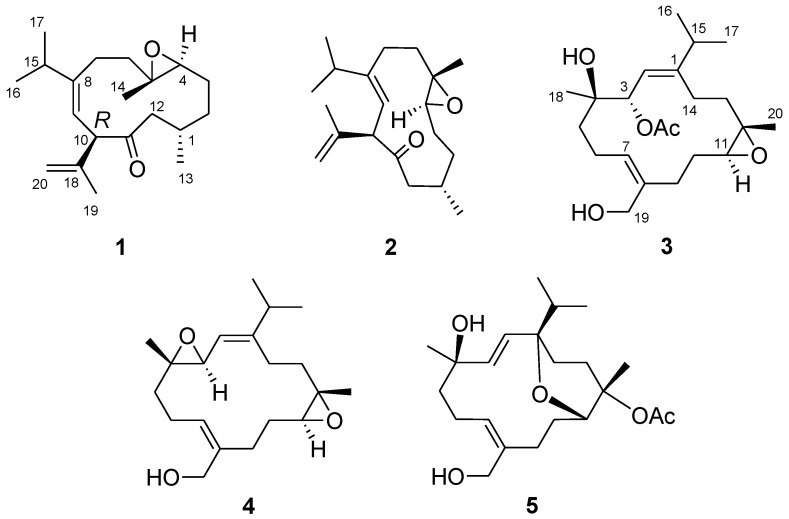
Structures of compounds **1**–**5**.

Our previous study on the soft coral *Sinularia nanolobata* (Verseveldt), collected off the coast of the most southern tip of Taiwan, resulted in the characterization of caryophyllane- and xeniaphyllane-type terpenoids [11]. Another collection of the same species off the coast of northern region of Taiwan led to the isolation of a novel C18 terpenoid [12]. Herein, we report the investigation on the chemical constituents of *S. nanolobata,* collected off the coast of the eastern region of Taiwan, which led to the isolation of two new cubitanoids, nanoculones A and B (**1** and **2**), three new cembranoids, nanolobols A–C (**3**–**5**) (Figure 1), as well as six known compounds (**6**‒**11**). The cytotoxicity of compounds **1**–**11** against cancer cell lines P388 (murine leukemia), K562 (human erythromyeloblastoid leukemia), and HT-29 (human colon adenocarcinoma) was evaluated. In addition, the nitric oxide inhibitory activities of compounds **1**–**4**, **6**–**8**, **10**, and **11** were further evaluated by assay of lipopolysaccharide (LPS)-stimulated NO production in activated RAW264.7 cells.

## 2. Results and Discussion

**Figure 2:**
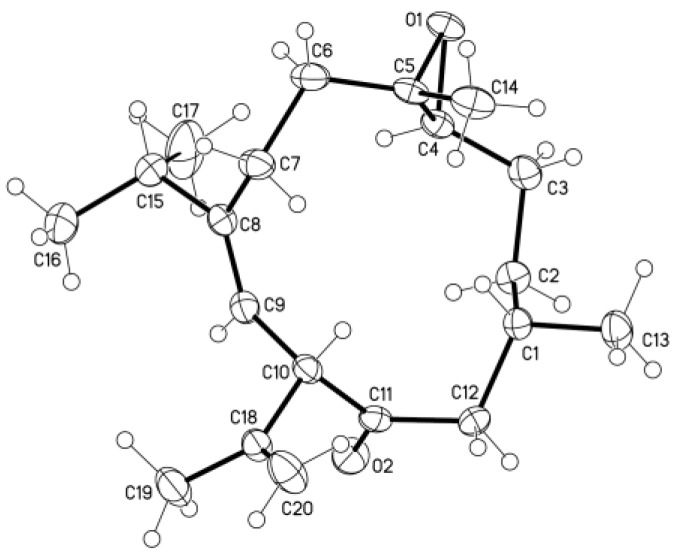
X-ray structure of **6**.

The EtOH extract of *S. nanolobata* was concentrated and partitioned with EtOAc, successively. The resulting EtOAc layer was separated repeatedly by column chromatography and High-performance liquid chromatography (HPLC) to afford five new diterpenoids (**1**–**5**) (Figure 1) and six known compounds (**6**–**11**). The known compounds were identified as calyculone I (**6**) [5], sinulariuol A (**7**) [13], sinulariols C, D, H, and J (**8**–**11**) [13], by comparison of their spectroscopic data with those reported in the literature. A single-crystal X-ray analysis was performed on **6**, which led to the establishment of the absolute configuration of **6** (Figure 2).

Inspection of the high resolution electrospray ionization mass spectroscopy (HRESIMS) (Supplementary Materials, Figure S1) and ^13^C nuclear magnetic resonance (NMR) spectroscopic data allowed the establishment of a molecular formula of C_20_H_32_O_2_ for **1**, requiring five degrees of unsaturation. The IR spectrum showed the presence of carbonyl group (1710 cm^−1^). The NMR spectra of **1** (Figures S2 and S3) displayed signals attributable to a ketone [δ_C_ 209.7 (qC)], a trisubstituted double bond [δ_C_ 147.1 (qC), δ_C_ 120.4 (CH); δ_H_ 5.45 (1H, d, *J* = 10.0 Hz)], a disubstituted double bond [δ_C_ 143.0 (qC), δ_C_ 112.6 (CH_2_); δ_H_ 4.85 and 4.80 (each 1H, s)], and a trisubstituted epoxide [δ_C_ 62.3 (qC), δ_C_ 62.5 (CH); δ_H_ 2.77 (1H, dd, *J* = 9.2, 3.6 Hz)] (Table 1), which accounted for four degrees of unsaturation and two oxygen atoms in the molecular formula. Thus, the structure of **1** should contain an additional ring. The above data, in conjunction with a highly deshielded proton at δ_H_ 4.21 (1H, d, *J* = 10.0 Hz, H-10) coupled by the olefinic H-9, were reminiscent of a cubitane skeleton [1,2,3,4,5].

**Figure 3:**
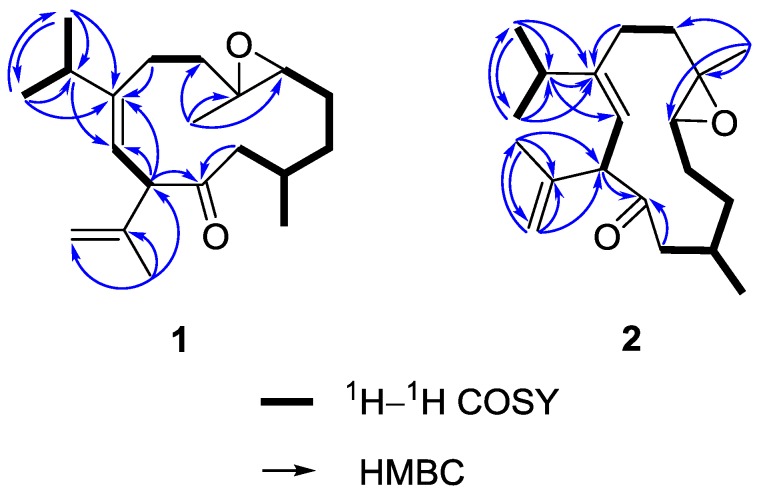
Selected ^1^H–^1^H COSY and HMBC correlations of **1** and **2**.

On the basis of H–H correlation spectroscopy (COSY) correlations of H_2_-12/H-1/H_2_-2/H_2_-3/H-4, H-1/H_3_-13, H_2_-6/H_2_-7, H_3_-16/H-15/H_3_-17, and H-9/H-10, the spin systems were established as shown in Figure 3. An isopropyl was deduced to be attached at C-8 according to the heteronuclear multiple bond correlation (HMBC) correlations from H_3_-16 (or H_3_-17) to C-15, C-17 (or C-16), and C-8. A propen-2-yl fragment was assigned at C-10 based on the HMBC correlations from H_3_-19 to C-10, C-18, and C-20. The methyl-substituted epoxide was assigned according to the HMBC correlations from H_3_-14 to C-4, C-5, and C-6. Accordingly, the planar structure of **1** was established and found to be the same as that of calyculone I (**6**) [5].

**Figure 4:**
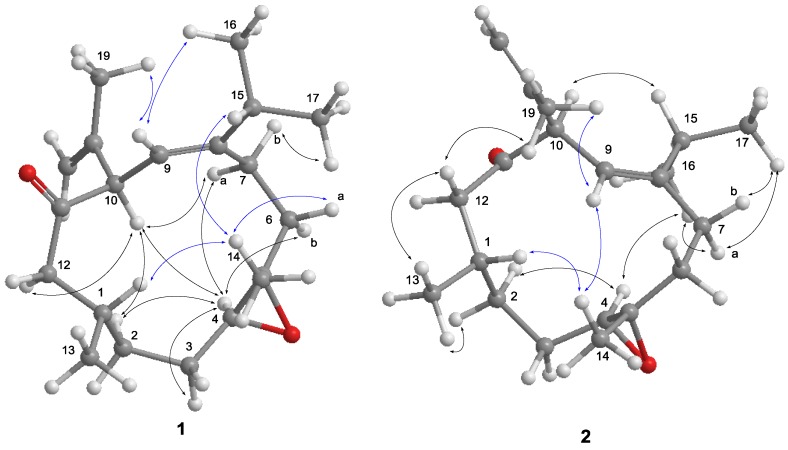
Selected NOE correlations of compounds **1** and **2**.

The nuclear Overhauser effect spectroscopy (NOESY) experiment was performed and its correlations were interpreted for the elucidation of the relative configuration of **1** (Figure 4). An NOE correlation between H-9 and H_3_-16 suggested an *E* geometry for C-8/C-9 double bond. The absence of an NOE cross peak between H_3_-14 and H-4 along with the presence of a correlation between H-4 and H-6b conducted to assign a *trans* geometry for 4,5-epoxide. The observed NOE correlations of H-10/H-4, H-4/H-7a and H-10/H-7a suggested the same orientation of H-10 and H-4 in the molecule, whereas a correlation of H_3_-14/H-1 disclosed that H-1 is oriented opposite H-10. Thus, **1** was deduced as a C-10 epimer of calyculone I (**6**).

Analysis of the ^13^C NMR and HRESIMS spectral data of **2** reveal the same molecular formula as that of **1** (Figures S4 and S6). The interpretation of NMR data and COSY and HMBC correlations (Figure 3) again showed that it is possibly a diastereomer or geometrical isomer of **1**. The NOE experiment allowed the relative configuration of **2** to be defined. As shown in Figure 4, the NOE correlation of H-10/H-15 as well as correlations of H-4/H_3_-16 and H_3_-16/H-7a conducted to assign an 8*Z* olefin and a *trans* epoxide. Moreover, correlations of H-1/H_3_-14 and H_3_-14/H-9 suggested that H-1 and H_3_-14 were oriented on opposite face to H-10. Consequently, **2** was determined as an 8*Z* epimer of **1**.

The HRESIMS of **3** displayed a sodiated molecular ion peak at *m/z* 403.2456 [M + Na]^+^ (Figure S7), indicative of a molecular formula of C_22_H_36_O_5_, suggesting five degrees of unsaturation. Its IR spectrum showed characteristic absorptions due to the presence of hydroxy (3437 cm^−1^) and ester carbonyl (1732 cm^−1^) groups. The latter was determined as an acetoxy functionality based on the NMR resonances at δ_C_ 170.9 (C), δ_C_ 21.4 (CH_3_), and δ_H_ 2.07 (Table 2). The ^13^C NMR spectrum (Figure S9) also showed characteristic signals composed of two olefins [δ_C_ 151.8 (C), 137.7 (C), 131.7 (CH), and 118.2 (CH)], an epoxide [δ_C_ 62.2 (CH) and 61.6 (C)], and three oxygen-bearing carbons [δ_C_ 74.8 (CH), 74.0 (C), and 59.5 (CH_2_)]. The above functionalities accounted for four of the five degrees of unsaturation, implying **3** to be monocyclic. Except for the acetyl group, its NMR data resemble those of sinulariol L [13], while 2D NMR interpretation revealed that **3** was a 3-*O*-acetyl analogue of sinulariol L. Analysis of NOE correlations of **3** ascertained that they shared the same relative configurations at C-3, C-4, C-11, and C-12, as well as the same geometries of C-1 and C-7 double bonds.

**Figure 5:**
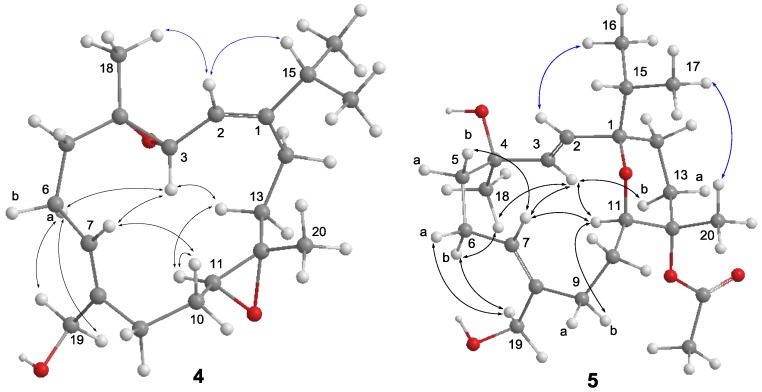
Selected NOE correlations of **4** and **5**.

The molecular formula of **4** was determined as C_20_H_32_O_3_ by the analysis of its HRESIMS and ^13^C NMR spectrum (Figures S10 and S12). Its NMR spectroscopic data (Table 2) also were found to be mostly similar to those of sinulariol L [13]. Inspection of ^1^H and ^13^C NMR data of **4** suggested the presence of two epoxides [δ_C_ 61.9 (CH), 61.3 (C × 2), and 59.5 (CH); δ_H_ 3.33 (1H, d, *J* = 6.8 Hz) and 2.73 (1H, dd, *J* = 10.0, 4.0 Hz)]. These data suggested **4** to be a 3,4-epoxide analogue of sinulariol L, which was corroborated by the HMBC correlations from H_3_-18 to C-3, C-4, and C-5 as well as COSY correlation between H-2 and H-3. The 1*E* and 7*Z* geometries were inferred according to the NOE correlations of H-2/H-15 and H_2_-19/H-6a, while correlations of H_3_-18/H-2 and H-11/H-13b suggested the *trans* geometries for both epoxides (Figure 5). The relative configurations at C-3, C-4, C-11, and C-12 were determined by further analysis of NOE correlations. The NOE correlations of H-3/H-6a, H-3/H-7, H-3/H-13b, and H-11/H-13b indicated the spatial proximity among these protons and, thus, a 3S*, 4S*, 11S*, 12S*-configuration was determined.

**Figure 6:**
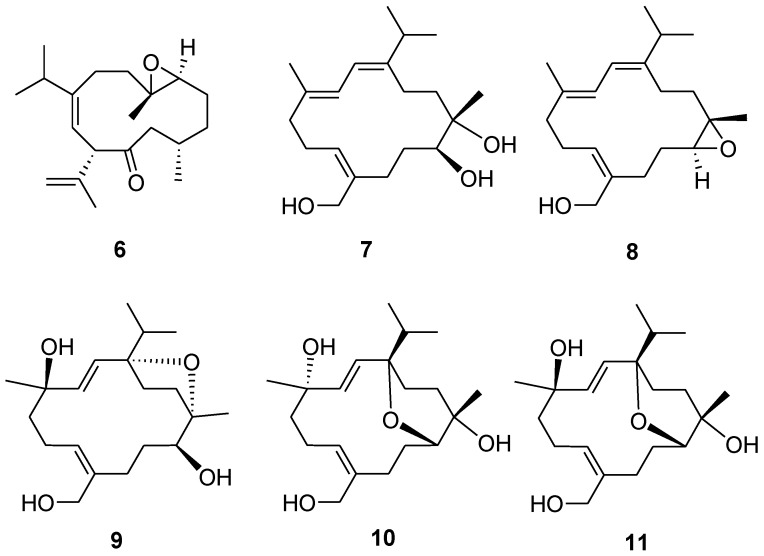
Structures of compounds **6**–**11**.

Compound **5** has a molecular formula of C_22_H_36_O_5_ as determined by HRESIMS and ^13^C NMR spectrum (Figures S13 and S15). The NMR data of **5** (Table 2) substantially resemble those of sinulariol J [13], except for the presence of an additional acetyl group featured resonances at δ_C_ 170.2 (C), δ_C_ 22.4 (CH_3_), and δ_H_ 1.98 (3H, s), suggesting **5** to be an *O*-acetyl analogue of sinulariol J. The downfield-shifted C-12 (δ_C_ 81.9 (C) and H_3_-20 (δ_H_ 1.44) and the HMBC correlations from H_3_-20 to C-11, C-12, and C-13 evidenced that this acetoxy group should be attached at C-12. The 2*E* and 7*Z* geometries were determined to be the same as those of sinulariol J according to the large coupling constant (*J* = 16.0 Hz) between H-2 and H-3, as well as NOE correlations of H_2_-19/H_2_-6 (Figure 5). In addition, NOE correlations of H-3/H-11 and H-3/H_3_-18 suggested they were oriented on the same face, and arbitrarily assigned as α-orientation. The isopropyl group and H_3_-20 were, thus, assigned as β-orientation based on the correlations of H-2/H_3_-16 and H_3_-17/H_3_-20. Consequently, an 1R*,4R*,11R*,12S*-configuration was assigned for **5**. Moreover, the NMR spectroscopic data of six known compounds **6**–**11** (Figure 6) were found to be identical to those of known compounds based on the comparison of their physical and spectroscopic data with those reported in the literature [5,13].

**Figure 7:**
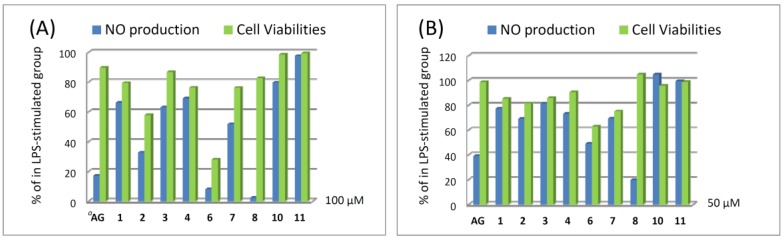
Nitric oxide (NO) production and cell viabilities of compounds **1**–**4**, **6**–**8**, **10**, and **11** in LPS-stimulated RAW264.7 cells (**A**) at 100 μM (**B**) at 50 μM. *^a^*AG: aminoguanidine is used as a positive control.

Compounds **1**–**11** were evaluated for cytotoxicity activities against P388, K562, and HT-29 cell lines using the Alamar Blue assay. The result showed that they are not cytotoxic against the above three cancer cell lines. In addition, the nitric oxide (NO) inhibitory activities of compounds **1**–**4**, **6**–**8**, **10**, and **11** were further evaluated by assay of LPS-stimulated NO production in activated RAW264.7 cells, as shown in Figure 7. The results indicated that compounds **2**, **6**, and **8** could effectively reduce the levels of NO to 32.6%, 8.0%, and 2.3%, respectively, at a concentration of 100 μM. Moreover, compound **8** at a concentration of 50 μM exhibited good inhibitory activity compared to the positive control aminoguanidine (AG), and the level of NO was also reduced significantly to 19.6%, while giving a 104.6% retention of cell viability. Thus, compound **8** is a promising metabolite which may become a lead compound in the future anti-inflammatory drug development.

## 3. Experimental Section

### 3.1. General Experimental Procedures

Optical rotations were measured on a JASCO P-1020 digital polarimeter (JASCO Corporation, Tokyo, Japan). IR spectra were recorded on a JASCO FT/IR-4100 infrared spectrophotometer (JASCO Corporation). The ^1^H NMR and ^13^C NMR spectra were recorded on a Varian 400MR NMR (400 MHz for ^1^H and 100 MHz for ^13^C) and a Varian Unity INOVA500 FT-NMR (500 MHz for ^1^H and 125 MHz for ^13^C) instruments (Varian Inc., Palo Alto, CA, USA). The chemical shifts were referenced to the solvent residue of CDCl_3_ (δ_H_ 7.265 ppm and δ_C_ 77.0 ppm). The high-resolution mass spectra were acquired via a Bruker APEX II mass spectrometer with an ESI ionization source (Bruker, Bremen, Germany). Silica gel 60 (40−63 μm, Merck, Darmstadt, Germany), C18 gel (LiChroprep RP-18, 40−63 μm, Merck), and Sephadex LH-20 (GE Healthcare, Uppsala, Sweden) were used for column chromatography. Thin-layer chromatography (TLC) analysis was performed on a precoated silica gel plates (Kieselgel 60 F_254_, 0.25 mm, Merck). High-performance liquid chromatography (HPLC) was performed using a Shimadzu LC-10AT*_VP_* series pump equipped with a UV detector (Shimadzu, Milan, Italy) and a semipreparative RP-18 column (5 µm, 250 × 10 mm, Hibar Purospher RP-18e, Merck).

### 3.2. Animal Material 

The soft coral *Sinularia nanolobata* was collected off the coast of Jihui Fishing Port, Taitung county, Taiwan (latitude: 23°07′ N; longitude: 121°23′ E) in March 2013. The material was frozen at −20 °C until extraction in the laboratory. Species identification of this coral was performed by Prof. C.-F. Dai (National Taiwan University, Taiwan), while the voucher specimen (JiH-201303) was deposited at the Department of Marine Biotechnology and Resources, National Sun Yat-sen University.

### 3.3. Extraction and Isolation

The minced, wet tissues of *S. nanolobata* (3.1 kg) were extracted with EtOH (3L × 2). The extract was concentrated under reduced pressure and the residue was partitioned between EtOAc and H_2_O. The EtOAc layer was dried over Na_2_SO_4_ and the resulting residue (27.9 g) was subjected to a silica gel column with a gradient of acetone and hexane in an increasing polarity (acetone-hexane, 2% to 100%), and then a gradient of MeOH and acetone (MeOH-acetone, 2% to 100%) to yield 18 fractions. Fraction 7 was chromatographed on a silica gel column with acetone/hexane (6%) to yield two subfractions 7a and 7b. Subfraction 7a was purified by a C18 gel column (MeOH-H_2_O, 90%), followed by semipreparative HPLC (MeOH-H_2_O, 78%) to yield compounds **1** (34.3 mg), **2** (12.0 mg) and **6** (12.6 mg). Fraction 10 was purified by a Sephadex LH-20 column using acetone as eluent to obtain a mixture, which was further separated by silica gel column chromatography (acetone-hexane, 10% to 100%), C18 column chromatography (MeOH-H_2_O, 90%), and semipreparative HPLC (MeOH-H_2_O, 83%) to yield compounds **4** (2.6 mg) and **8** (5.5 mg). Fraction 14 was purified by a Sephadex LH-20 column (acetone, 100%) and semipreparative HPLC (MeOH-H_2_O, 80%) to yield compounds **3** (5.2 mg) and **5** (1.2 mg), and (MeOH-H_2_O, 67%) to yield compounds **7** (3.8 mg) and **9** (6.4 mg). Fraction 15 was purified by a Sephadex LH-20 column using acetone as eluent to obtain a mixture, which was further separated by silica gel column chromatography (acetone-hexane, 20% to 100%), C18 column chromatography (MeOH-H_2_O, 90%), and semipreparative HPLC (MeOH-H_2_O, 66%) to yield compounds **10** (2.9 mg) and **11** (2.6 mg).

Nanoculone A (**1**): colorless oil; ${\left[\alpha \right]}_{D}^{25}$ +6 (*c* 1.20, CHCl_3_); IR (neat) ν_max_ 2962, 1710, 1644, 1457, 1379, and 895 cm^−1^; ^13^C and ^1^H NMR data, see Table 1; ESIMS *m/z* 327 [M + Na]^+^; HRESIMS *m/z* 327.2298 [M + Na]^+^ (calcd. for C_20_H_32_O_2_Na, 327.2300).

Nanoculone B (**2**): colorless oil; ${\left[\alpha \right]}_{D}^{25}$ +175 (*c* 0.49, CHCl_3_); IR (neat) ν_max_ 2957, 2866, 1705, 1455, 1105, and 895 cm^−1^; ^13^C and ^1^H NMR data, see Table 1; ESIMS *m/z* 327 [M + Na]^+^; HRESIMS *m/z* 327.2297 [M + Na]^+^ (calcd. for C_20_H_32_O_2_Na, 327.2300).

Nanolobol A (**3**): colorless oil; ${\left[\alpha \right]}_{D}^{25}$ +3 (*c* 1.30, CHCl_3_); IR (neat) ν_max_ 3437, 2957, 2960, 1732, 1459, 1372, 1246, 1020, and 756 cm^−1^; ^13^C and ^1^H NMR data, see Table 2; ESIMS *m/z* 403 [M + Na]^+^; HRESIMS *m/z* 403.2456 [M + Na]^+^ (calcd. for C_22_H_36_O_5_Na, 403.2455).

Nanolobol B (**4**): colorless oil; ${\left[\alpha \right]}_{D}^{25}$ −67 (*c* 0.65, CHCl_3_); IR (neat) ν_max_ 3441, 2923, 2960, 1731, 1462, 1024, and 856 cm^−1^; ^13^C and ^1^H NMR data, see Table 2; ESIMS *m/z* 343 [M + Na]^+^; HRESIMS *m/z* 343.2244 [M + Na]^+^ (calcd. for C_20_H_32_O_3_Na, 343.2244).

Nanolobol C (**5**): colorless oil; ${\left[\alpha \right]}_{D}^{25}$ −86 (*c* 0.20, CHCl_3_); IR (neat) ν_max_ 3384, 2959, 2874, 1724, 1601, 1438, 1367, 1256, and 1095 cm^−1^; ^13^C and ^1^H NMR data, see Table 2; ESIMS *m/z* 403 [M + Na]^+^; HRESIMS *m/z* 403.2456 [M + Na]^+^ (calcd. for C_22_H_36_O_5_Na, 403.2455).

Calyculone I (**6**): colorless needles; ${\left[\alpha \right]}_{D}^{25}$ +41 (*c* 1.09, CHCl_3_); lit. ${\left[\alpha \right]}_{D}^{20}$ +52.1 (c 1.0, CHCl_3_) [5]; IR (neat) ν_max_ 2959, 1708, 1463, 1385 and 897 cm^−1^; ESIMS *m/z* 455 [M + Na]^+^ (calcd. for C_20_H_32_O_2_Na).

Sinulariol A (**7**): colorless oil; ${\left[\alpha \right]}_{D}^{21}$ +47 (*c* 0.95, CHCl_3_); lit. ${\left[\alpha \right]}_{D}^{24}$ +17.1 (c 0.79, CHCl_3_) [13]; IR (neat) ν_max_ 3369, 2959, 2871, 1447, 1379 and 756 cm^−1^; ESIMS *m/z* 345 [M + Na]^+^ (calcd. for C_20_H_34_O_3_Na).

Sinulariol C (**8**): colorless oil; ${\left[\alpha \right]}_{D}^{25}$ −30 (*c* 0.31, CHCl_3_); lit. ${\left[\alpha \right]}_{D}^{22}$ −8.8 (c 0.15, CHCl_3_) [13]; IR (neat) ν_max_ 3421, 2960, 1671, 1458, 1384, 1021, 857, 755 and 669 cm^−1^; ESIMS *m/z* 327 [M + Na]^+^ (calcd. for C_20_H_32_O_2_Na).

Sinulariol D (**9**): colorless oil; ${\left[\alpha \right]}_{D}^{25}$ +13 (*c* 0.46, CHCl_3_); lit. ${\left[\alpha \right]}_{D}^{24}$ +7.5 (c 1.35, CHCl_3_) [13]; IR (neat) ν_max_ 3355, 2963, 2873, 1653, 1455, 1367, 1077 and 756 cm^−1^; ESIMS *m/z* 361 [M + Na]^+^ (calcd. for C_20_H_34_O_4_Na).

Sinulariol H (**10**): colorless oil; ${\left[\alpha \right]}_{D}^{21}$ −78 (*c* 0.73, CHCl_3_); lit. ${\left[\alpha \right]}_{D}^{24}$ −71.3 (c 0.28, CHCl_3_) [13]; IR (neat) ν_max_ 3371, 2963, 2933, 1444, 1374 and 756 cm^−1^; ESIMS *m/z* 361 [M + Na]^+^ (calcd. for C_20_H_34_O_4_Na).

Sinulariol J (**11**): colorless oil; ${\left[\alpha \right]}_{D}^{21}$ −77 (*c* 0.65, CHCl_3_); lit. ${\left[\alpha \right]}_{D}^{23}$ −40.5 (c 0.15, CHCl_3_) [13]; IR (neat) ν_max_ 3350, 2959, 2871, 1447, 1379 and 756 cm^−1^; ESIMS *m/z* 361 [M + Na]^+^ (calcd. for C_20_H_34_O_4_Na).

### 3.4. Crystallographic Data of **6**

The colorless crystal size of 0.15 × 0.10 × 0.10 mm was obtained at 4 °C in a refrigerator by slow evaporation in an acetone solution. Diffraction intensity data were acquired with a CCD area detector with graphite-monochromated Cu Kα radiation (λ = 1.54178 Å). Crystal data for **6**: C_20_H_32_O_2_, M = 304.45, monoclinic, *a* = 8.4794 (6) Å, *b* = 11.0386 (8) Å, *c* = 10.2704 (7) Å, *α* = 90°, *β* = 104.094 (3)°, *γ* = 90°, *V* = 932.38 (11) Å3, *T* = 100 (2) K, space group *P2_1_*, *Z* = 2, *μ* (CuKα) = 0.521 mm^−1^, 6301 reflections collected, 3100 independent reflections (*R_int_* = 0.0201). The final *R*_1_ values were 0.0275 (*I* > 2*σ*(*I*)). The final *wR* (*F*_2_) values were 0.0733 (*I* > 2*σ*(*I*)). The final *R*_1_ values were 0.0276 (all data). The final *wR* (*F*_2_) values were 0.0734 (all data). The goodness of fit on *F*_2_ was 1.050. Flack parameter = 0.1 (2). Crystallographic data for **6** have been deposited with the Cambridge Crystallographic Data Centre (deposition number CCDC 1482769). Copies of the data can be obtained, free of charge, on application to the Director, CCDC, 12 Union Road, Cambridge CB21EZ, UK.

### 3.5. Cytotoxicity Assay

The Alamar Blue assay were performed as previous reported [14,15]. After the cell lines (P388, K562, and HT-29) were cultured for 15 h according to the published procedure [16], the tested compounds in DMSO solutions were added and cultured for 72 h. The attached cells were incubated with Alamar Blue (10 μL/well, 4 h) and the absorbance was measured at wavelength of 595 nm using a microplate reader.

### 3.6. Nitric Oxide Inhibitory Activity

The nitrite concentration in the culture medium was measured as an indicator of NO production according to the Griess reaction [17]. Briefly, 80 μL of cell culture supernatant was reacted with 100 μL of Griess reagent (1:1 mixture of 0.1% *N*-(1-naphthyl)ethylenediamine dihydrochloride in water and 1% sulfanilamide in 5% phosphoric acid) in a 96-well plate and incubated at room temperature for 10 min. The absorbance at 550 nm was recorded using the ELISA reader [18,19]. Fresh medium was used as the blank. The results are expressed as the percentage of inhibition calculated relative to the cells treated with vehicle and LPS.

## 4. Conclusions

The prior studies demonstrated that the cubitanoids originating from marine organisms were elaborated as a minor group in certain species, such as gorgonian corals, *Eunicea calyculata* [3,4] and *E. laciniata* [20], and soft corals, *Sinularia triangula* [21,22] and *S. crassa* [23]. Our present study discovered new cubitanoids from the *S. nanolobata,* collected off the coast of the eastern region of Taiwan. Considering our prior work on the same species collected in different locations of Taiwan water [11,12], it is noteworthy that the metabolites from soft corals, even the skeletons, might vary with their geographical location.

## Figures and Tables

**Table 1:** ^1^H and ^13^C NMR spectroscopic data of compounds **1** and **2**.

Position	1		2	
	δ_H_ (*J* in Hz) *^a^*	δ_C_ (mult.) *^b^*	δ_H_ (*J* in Hz) *^a^*	δ_C_ (mult.) *^b^*
1	1.95 m	27.8, CH	2.06 m	29.2, CH
2	1.54 m	33.9, CH_2_	1.48 m	30.3, CH_2_
			1.24 m	
3	1.98 m	25.9, CH_2_	1.93 m	24.9, CH_2_
	1.30 m		1.24 m	
4	2.77 dd (9.2, 3.6)	62.5, CH	2.61 dd (10.0, 2.8)	64.6, CH
5		62.3, C		60.2, C
6	2.30 m	35.7, CH_2_	2.06 m	35.1, CH_2_
	1.39 m		1.62 m	
7	2.58 m	25.9, CH_2_	2.21 m	27.5, CH_2_
	2.10 m		2.09 m	
8		147.1, C		147.7, C
9	5.45 d (10.0)	120.4, CH	5.20 d (10.8)	119.3, CH
10	4.21 d (10.0)	57.7, CH	4.08 d (10.8)	60.5, CH
11		209.7, C		208.7, C
12	2.63 dd (14.8, 6.4)	52.6, CH_2_	2.54 dd (11.6, 2.8)	48.4, CH_2_
	2.28 m		1.68 m	
13	0.89 d (6.4)	20.6, CH_3_	0.95 d (6.4)	20.7, CH_3_
14	1.16 s	19.0, CH_3_	1.31, s	18.0, CH_3_
15	2.40 m	30.3, CH	2.87 m	29.6, CH
16	1.08 d (6.4)	20.5, CH_3_	1.07 d (7.0)	21.7, CH_3_
17	0.98 d (6.4)	23.0, CH_3_	1.01 d (7.0)	21.4, CH_3_
18		143.0, C		147.7, C
19	1.70 s	21.2, CH_3_	1.75 s	22.2, CH_3_
20	4.85 s	112.6, CH_2_	4.99 s	112.7, CH_2_
	4.80 s		4.90 s	

*^a^* Spectra recorded at 400 MHz in CDCl_3_; *^b^* Spectra recorded at 100 MHz in CDCl_3_.

**Table 2:** ^1^H and ^13^C NMR spectroscopic data of **3**–**5**.

Position	3		4		5	
	δ_H_ (*J* in Hz) *^a^*	δ_C_ (mult.) *^b^*	δ_H_ (*J* in Hz) *^a^*	δ_C_ (mult.) *^b^*	δ_H_ (*J* in Hz) *^c^*	δ_C_ (mult.) *^d^*
1		151.8, C		151.8, C		78.3, C
2	5.09 d (10.0)	118.2, CH	4.99 d (6.8)	119.2, CH	5.67 d (16.0)	126.8, CH
3	5.49 d (10.0)	74.8, CH	3.33 d (6.8)	59.5, CH	6.01 d (16.0)	140.8, CH
4		74.0, C		61.3, C		73.5, C
5	1.70 m	36.6, CH_2_	2.08 m	38.0, CH_2_	1.96 m	43.6, CH_2_
	1.59 m		1.47 m		1.68 m	
6	2.61 m	23.6, CH_2_	2.31 m	22.0, CH_2_	2.35 m	23.6, CH_2_
	2.12 m		2.09 m		2.21 m	
7	5.47 t (7.6)	131.7, CH	5.48 t (7.6)	129.9, CH	5.32 t (7.5)	134.2, CH
8		137.7, C		137.6, C		135.0, C
9	2.34 m	34.7, CH_2_	2.61 m	32.6, CH_2_	2.33 m	34.6, CH_2_
	2.23 m		2.05 m		2.12 m	
10	2.15 m	24.6, CH_2_	2.14 m	24.8, CH_2_	1.66 m	27.3, CH_2_
	1.44 m		1.37 m		1.52 m	
11	3.20 dd (9.6, 2.8)	62.2, CH	2.73 dd (10.0, 4.0)	61.9, CH	3.61 dd (8.0, 1.5)	72.6, CH
12		61.6, C		61.3, C		81.9, C
13	2.10 m	40.1, CH_2_	2.17 m	38.3, CH_2_	2.46 dt (11.5, 3.5)	31.5, CH_2_
	0.98 m		1.19 m		1.91 m	
14	2.30 m	27.0, CH_2_	2.17 m	27.4, CH_2_	1.86 m	26.4, CH_2_
	1.97 m				1.49 m	
15	2.34 m	35.3, CH	2.27 m	34.0, CH	1.85 m	39.1, CH
16	1.03 d (6.8)	21.8, CH_3_	1.03 d (6.8)	21.8, CH_3_	0.77 d (7.0)	17.2, CH_3_
17	1.03 d (6.8)	22.1, CH_3_	1.04 d (6.8)	22.3, CH_3_	0.92 d (7.0)	16.5, CH_3_
18	1.13 s	23.9, CH_3_	1.25 s	18.0, CH_3_	1.30 s	29.4, CH_3_
19	4.36 d (12.8)	59.5, CH_2_	4.29 d (12.0)	58.8, CH_2_	4.24 d (12.0)	60.2, CH_2_
	4.01 d (12.8)		4.18 d (12.0)		4.10 d (12.0)	
20	1.28 s	16.3 CH_3_	1.29 s	16.5 CH_3_	1.44 s	16.8 CH_3_
OAc		170.9, C				170.2, C
	2.07 s	21.4, CH_3_			1.98 s	22.4, CH_3_

*^a^* Spectra recorded at 400 MHz in CDCl_3_; *^b^* Spectra recorded at 100 MHz in CDCl_3_; *^c^* Spectra recorded at 500 MHz in CDCl_3_; *^d^* Spectra recorded at 125 MHz in CDCl_3_.

## Supplementary Materials

HRESIMS, ^1^H, and ^13^C spectra of new compounds **1**–**5** are available online at www.mdpi.com/1660-3397/14/8/150/s1. Figure S1: HRESIMS spectrum of **1**, Figure S2: ^1^H NMR spectrum of **1** in CDCl_3_ at 400 MHz, Figure S3: ^13^C NMR spectrum of **1** in CDCl_3_ at 100 MHz, Figure S4: HRESIMS spectrum of **2**, Figure S5: ^1^H NMR spectrum of **2** in CDCl_3_ at 400 MHz, Figure S6: ^13^C NMR spectrum of **2** in CDCl_3_ at 100 MHz, Figure S7: HRESIMS spectrum of **3**, Figure S8: ^1^H NMR spectrum of **3** in CDCl_3_ at 400 MHz, Figure S9: ^13^C NMR spectrum of **3** in CDCl_3_ at 100 MHz, Figure S10: HRESIMS spectrum of **4**, Figure S11: ^1^H NMR spectrum of **4** in CDCl_3_ at 400 MHz, Figure S12: ^13^C NMR spectrum of **4** in CDCl_3_ at 100 MHz, Figure S13: HRESIMS spectrum of **5**, Figure S14: ^1^H NMR spectrum of **5** in CDCl_3_ at 500 MHz, Figure S15: ^13^C NMR spectrum of **5** in CDCl_3_ at 125 MHz.
