# Supplementary material for: Cubitanoids and Cembranoids from the Soft Coral Sinularia nanolobata
Source: Mar Drugs. 2016 Aug 9;14(8):150. doi: 10.3390/md14080150 (PMC4999911; doi:10.3390/md14080150)
Supplement: Supplementary file 1 [file marinedrugs-14-00150-s001.pdf]

Supplementary Materials: Cubitanoids and Cembranoids from the Soft Coral *Sinularia nanolobata*

Chih-Hua Chao, Chia-Yun Wu, Chiung-Yao Huang, Hui-Chun Wang, Chang-Feng Dai, Yang-Chang Wu and Jyh-Horng Sheu

List of Supplementary material:

No	Content	page
**Figure S1**	HRESIMS spectrum of **1**.	S2
**Figure S2**	^{1}H NMR spectrum of **1** in $CDCl_3$ at 400 MHz.	S3
**Figure S3**	^{13}C NMR spectrum of **1** in $CDCl_3$ at 100 MHz.	S4
**Figure S4**	HRESIMS spectrum of **2**.	S5
**Figure S5**	^{1}H NMR spectrum of **2** in $CDCl_3$ at 400 MHz.	S6
**Figure S6**	^{13}C NMR spectrum of **2** in $CDCl_3$ at 100 MHz.	S7
**Figure S7**	HRESIMS spectrum of **3**.	S8
**Figure S8**	^{1}H NMR spectrum of **3** in $CDCl_3$ at 400 MHz.	S9
**Figure S9**	^{13}C NMR spectrum of **3** in $CDCl_3$ at 100 MHz.	S10
**Figure S10**	HRESIMS spectrum of **4**.	S11
**Figure S11**	^{1}H NMR spectrum of **4** in $CDCl_3$ at 400 MHz.	S12
**Figure S12**	^{13}C NMR spectrum of **4** in $CDCl_3$ at 100 MHz.	S13
**Figure S13**	HRESIMS spectrum of **5**.	S14
**Figure S14**	^{1}H NMR spectrum of **5** in $CDCl_3$ at 500 MHz.	S15
**Figure S15**	^{13}C NMR spectrum of **5** in $CDCl_3$ at 125 MHz.	S16

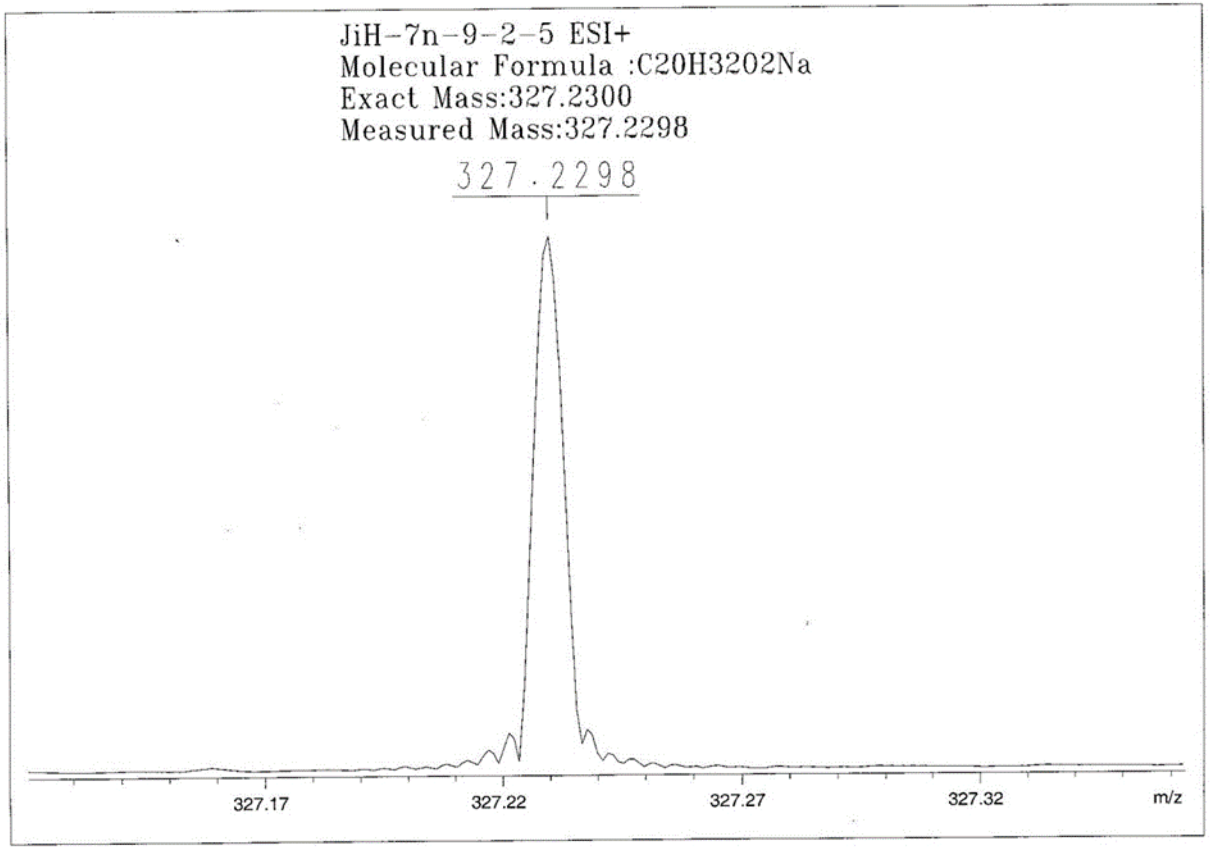

Figure S1. HRESIMS spectrum of **1**.

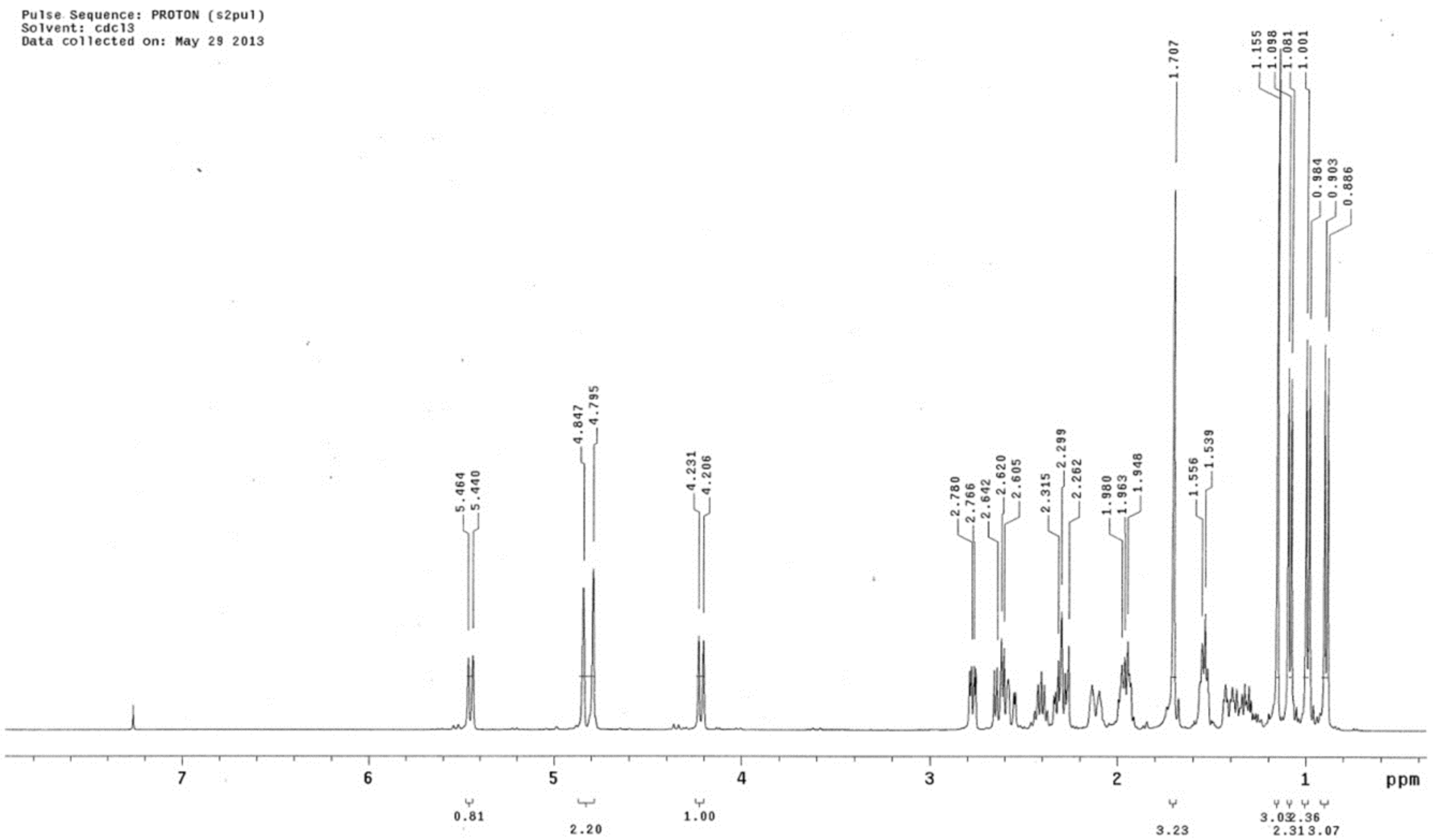

Figure S2. ^1H NMR spectrum of **1** in $CDCl_3$ at 400 MHz.

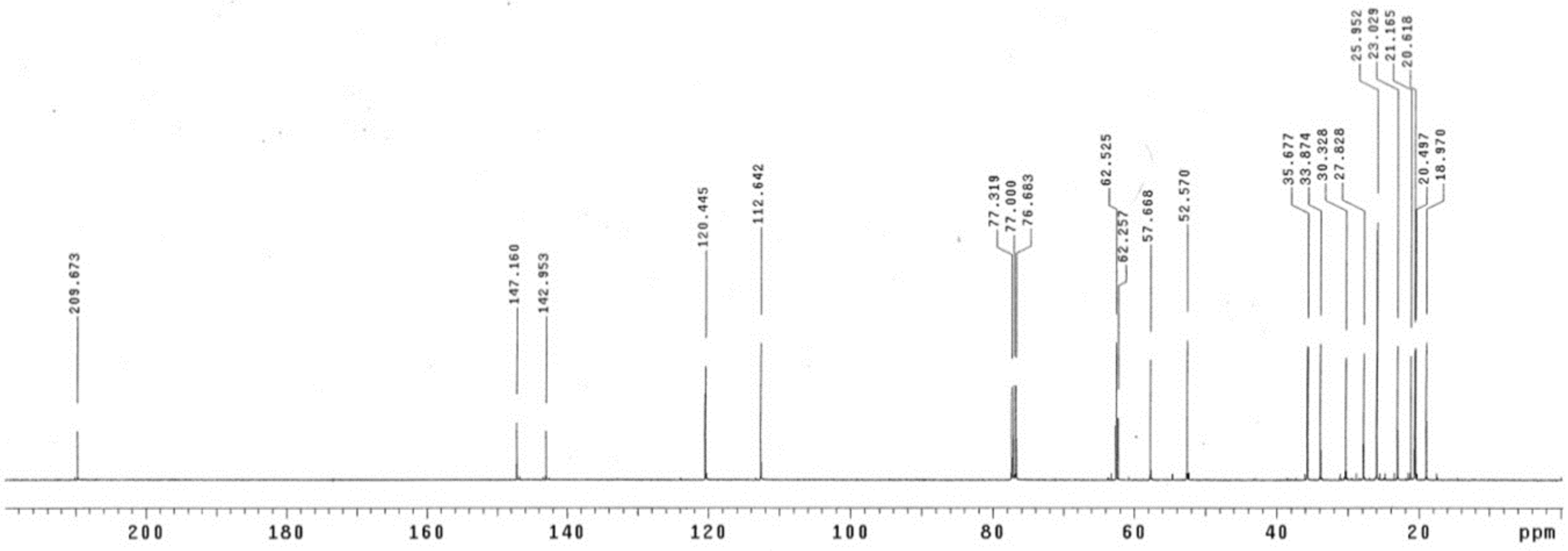

Figure S3. ^{13}C NMR spectrum of **1** in $CDCl_3$ at 100 MHz.

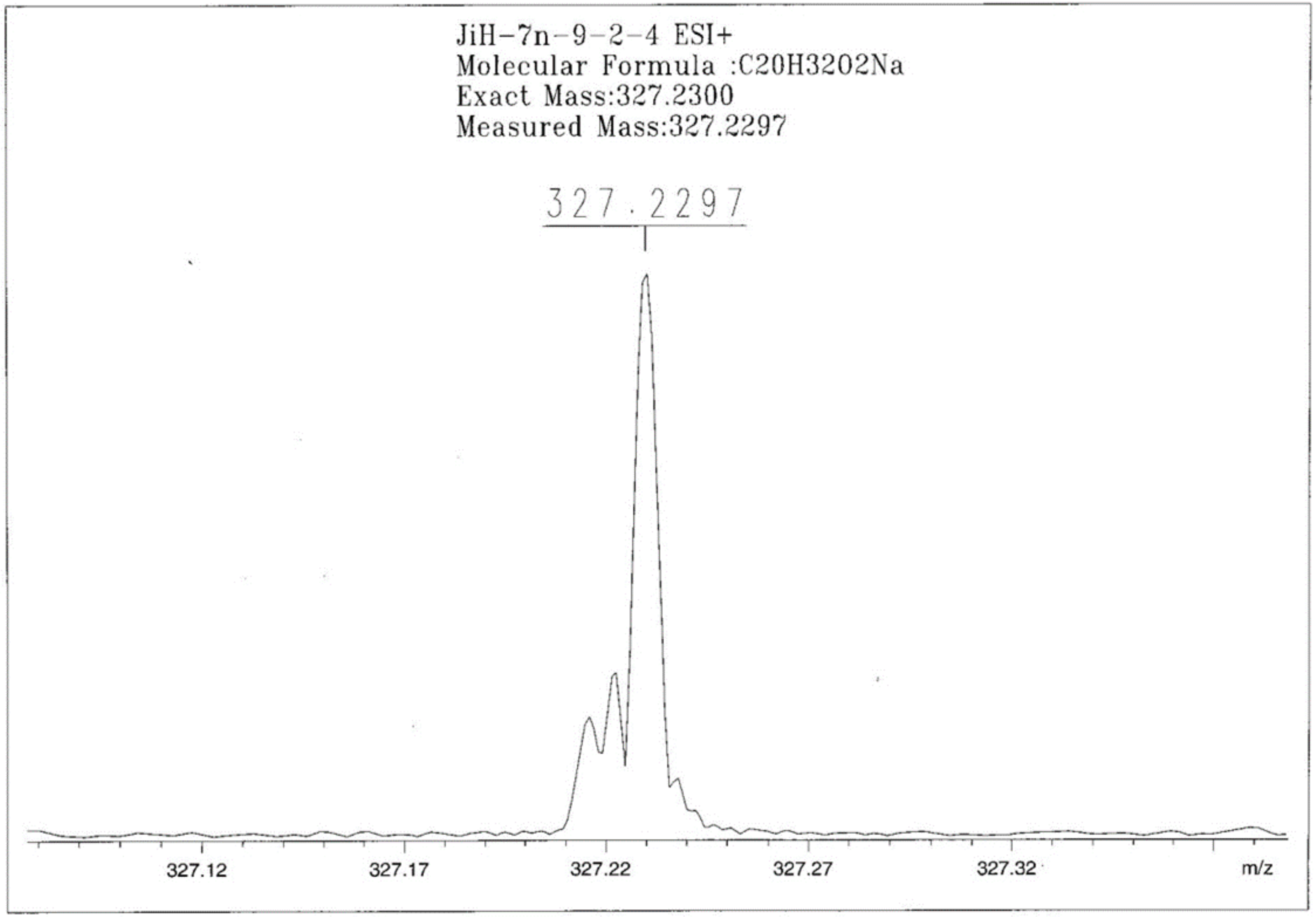

Figure S4. HRESIMS spectrum of **2**.

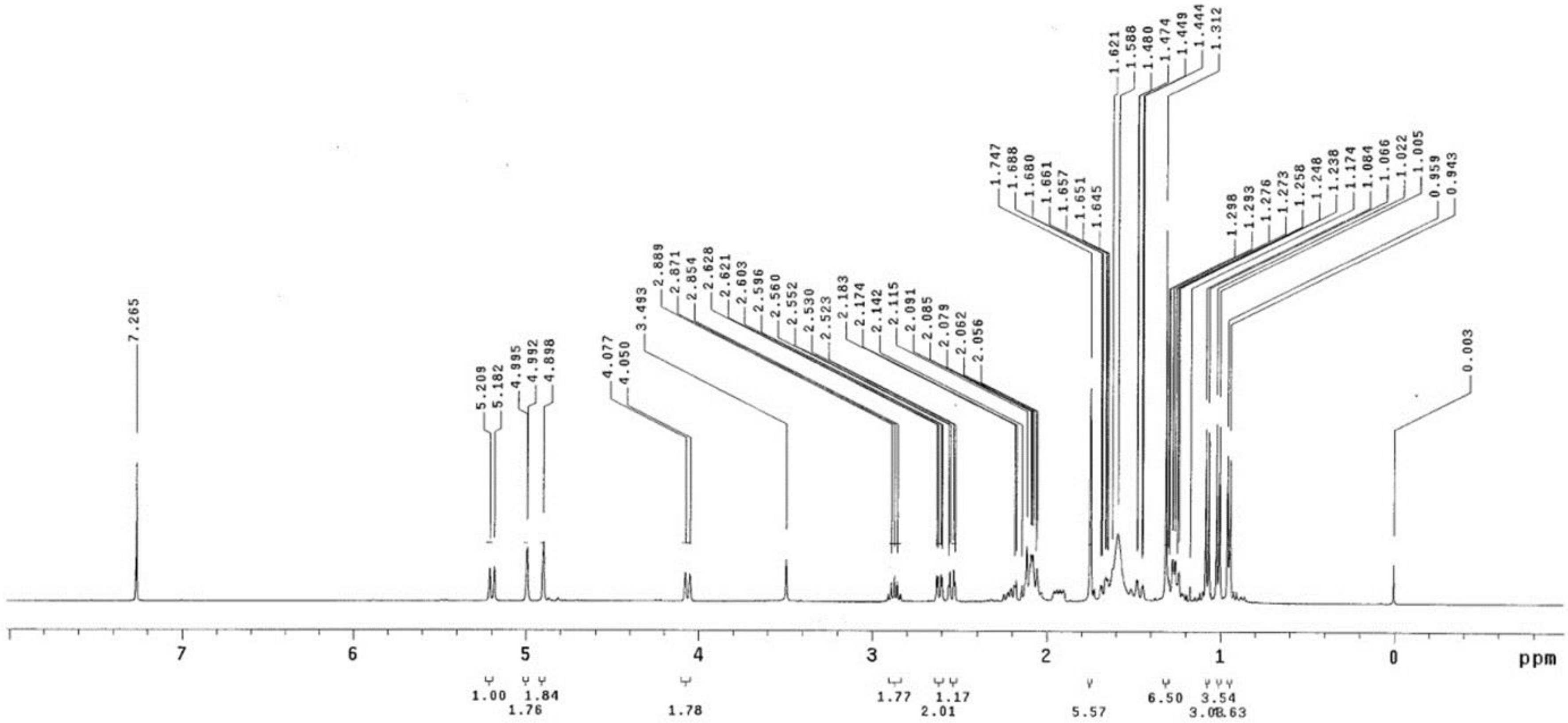

Figure S5. ^{1}H NMR spectrum of **2** in $CDCl_3$ at 400 MHz.

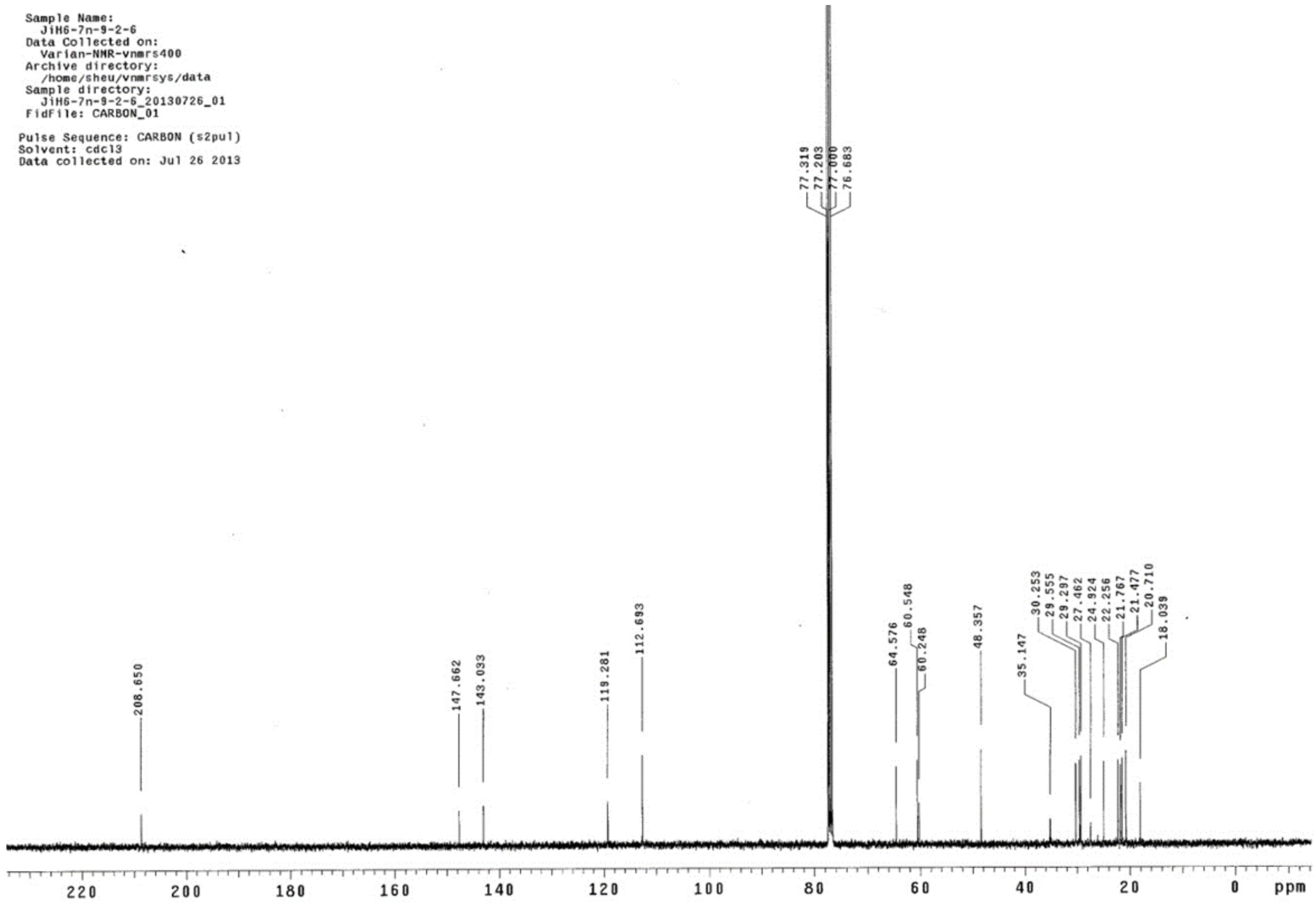

Figure S6. ^{13}C NMR spectrum of **2** in $CDCl_3$ at 100 MHz.

Mass Spectrum SmartFormula Report

Analysis Info

Analysis Name	D:\Data\a1\jih614n6531_000003.d	12/27/2013 4:17:13 PM
Method	broadband first signal	
Sample Name	JiH6-14n-6-5-3-1	Instrument: FT-MS solariX
Comment	ESI Positive	

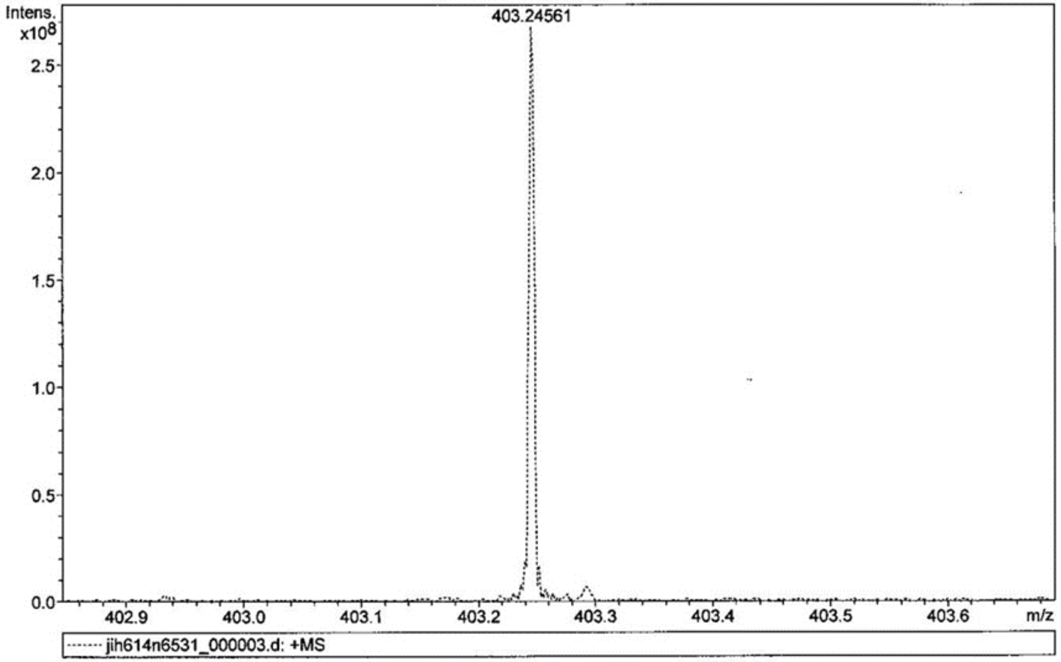

Meas. m/z	#	Formula	Score	m/z	err [mDa]	err [ppm]	mSigma	rdb	e⁻ Conf	N-Rule
403.24561	1	C 22 H 36 Na O 5	100.00	403.24550	-0.11	-0.28	10.8	4.5	even	ok

Figure S7. HRESIMS spectrum of **3**.

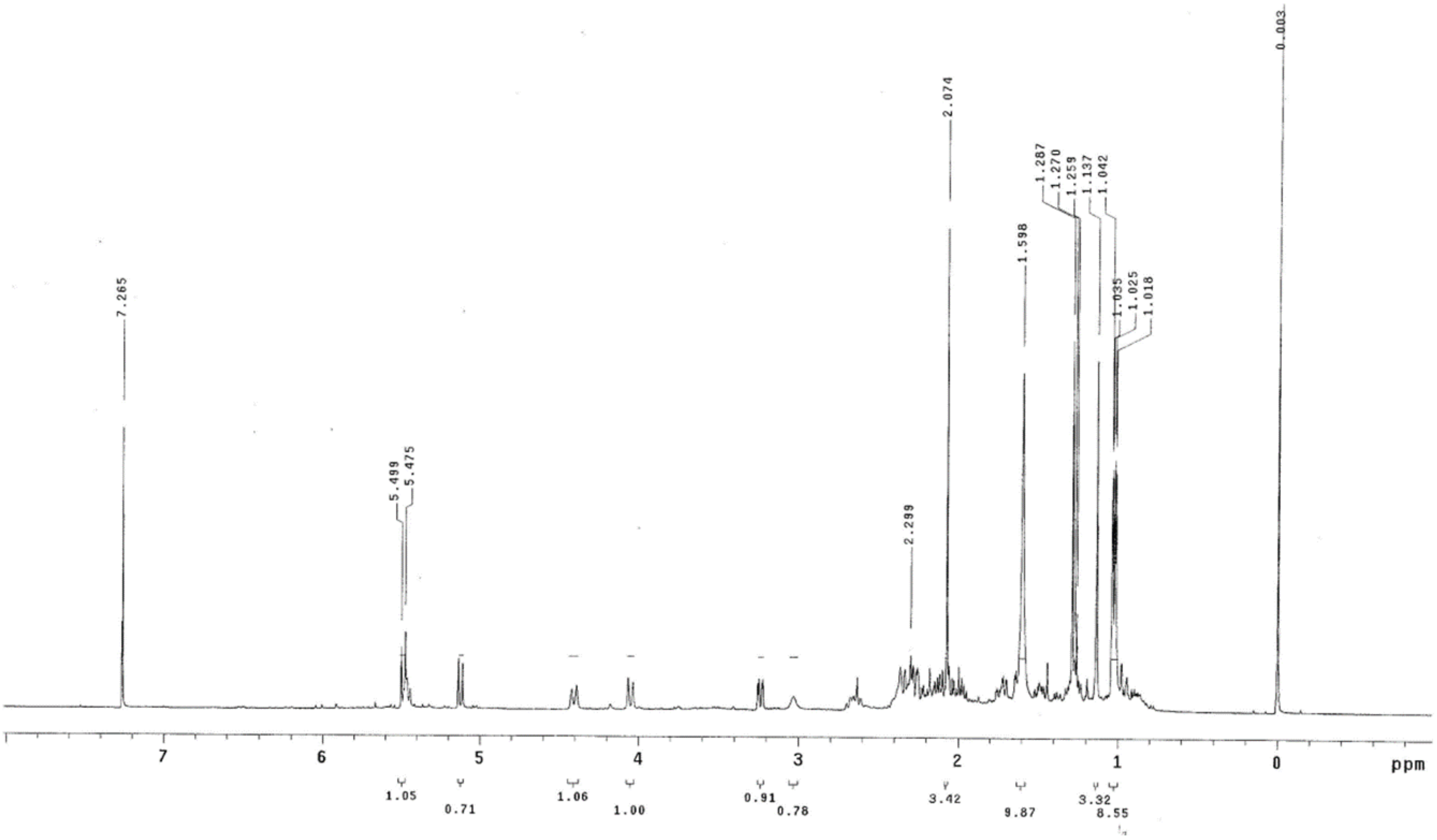

Figure S8. ^{1}H NMR spectrum of **3** in $CDCl_3$ at 400 MHz.

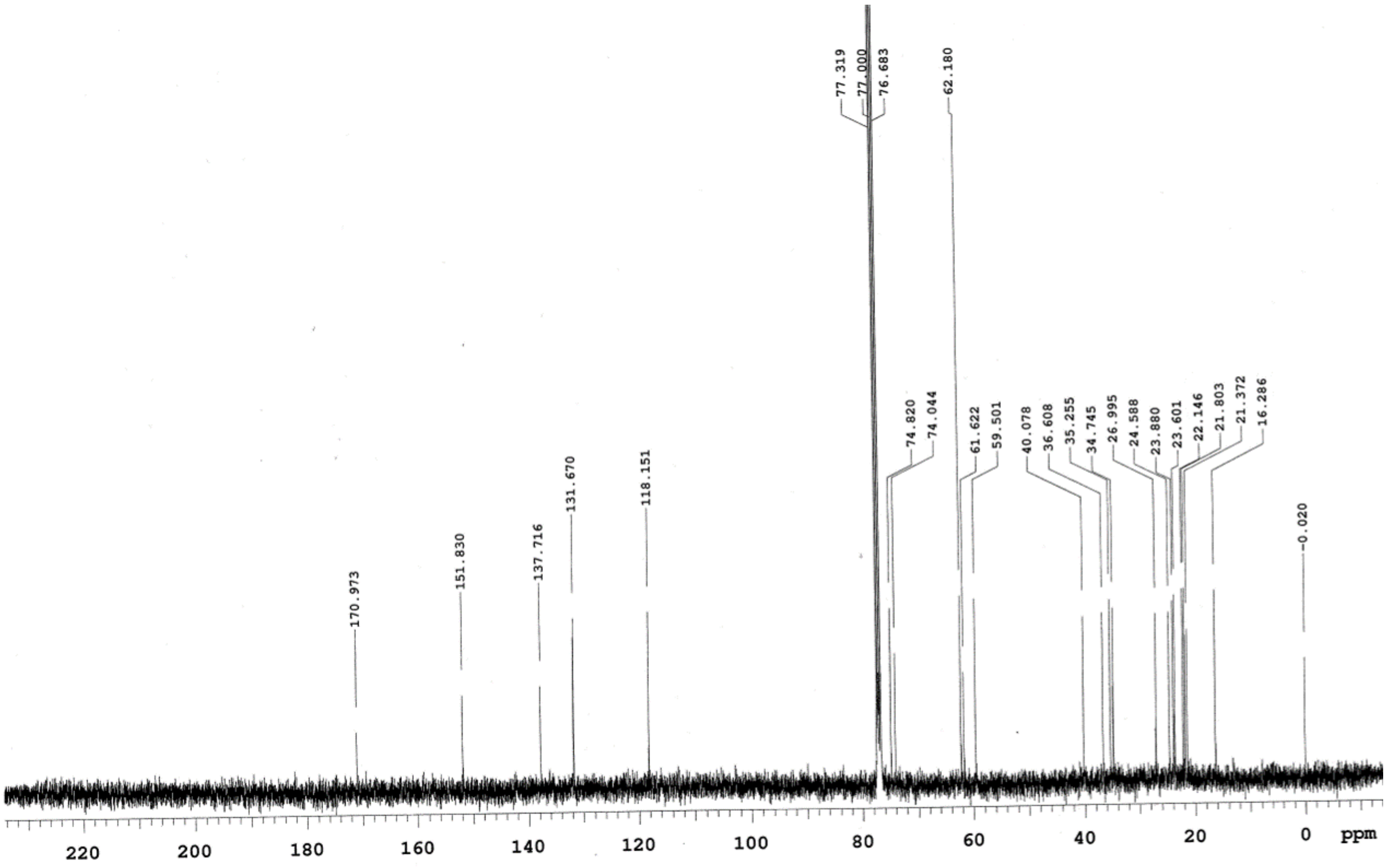

Figure S9. ^{13}C NMR spectrum of **3** in $CDCl_3$ at 100 MHz.

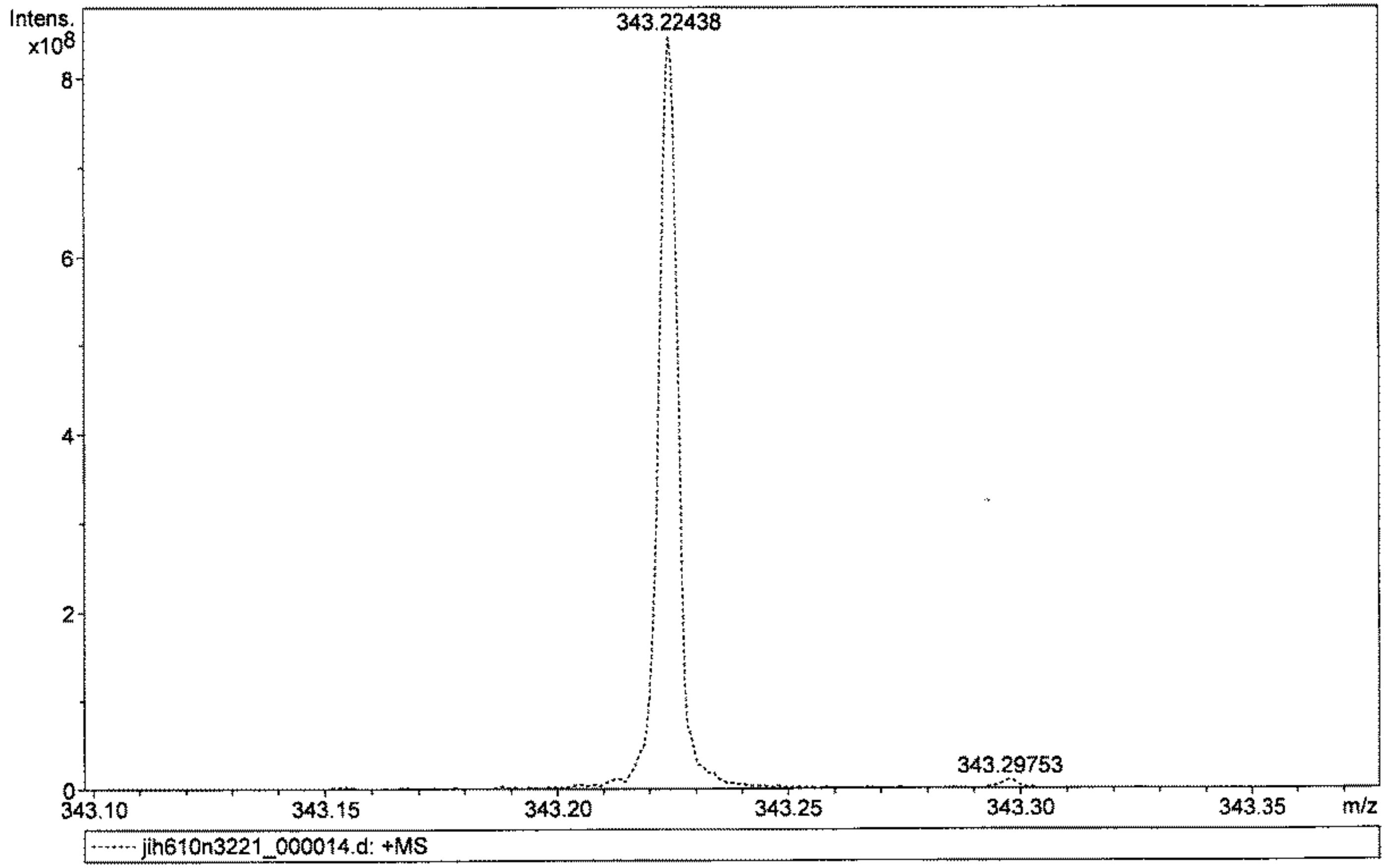

Meas. m/z	#	Formula	Score	m/z	err [mDa]	err [ppm]	mSigma	rdb	e⁻ Conf	N-Rule
343.22438	1	C 20 H 32 Na O 3	100.00	343.22437	-0.02	-0.05	8.2	4.5	even	ok

Figure S10. HRESIMS spectrum of **4**.

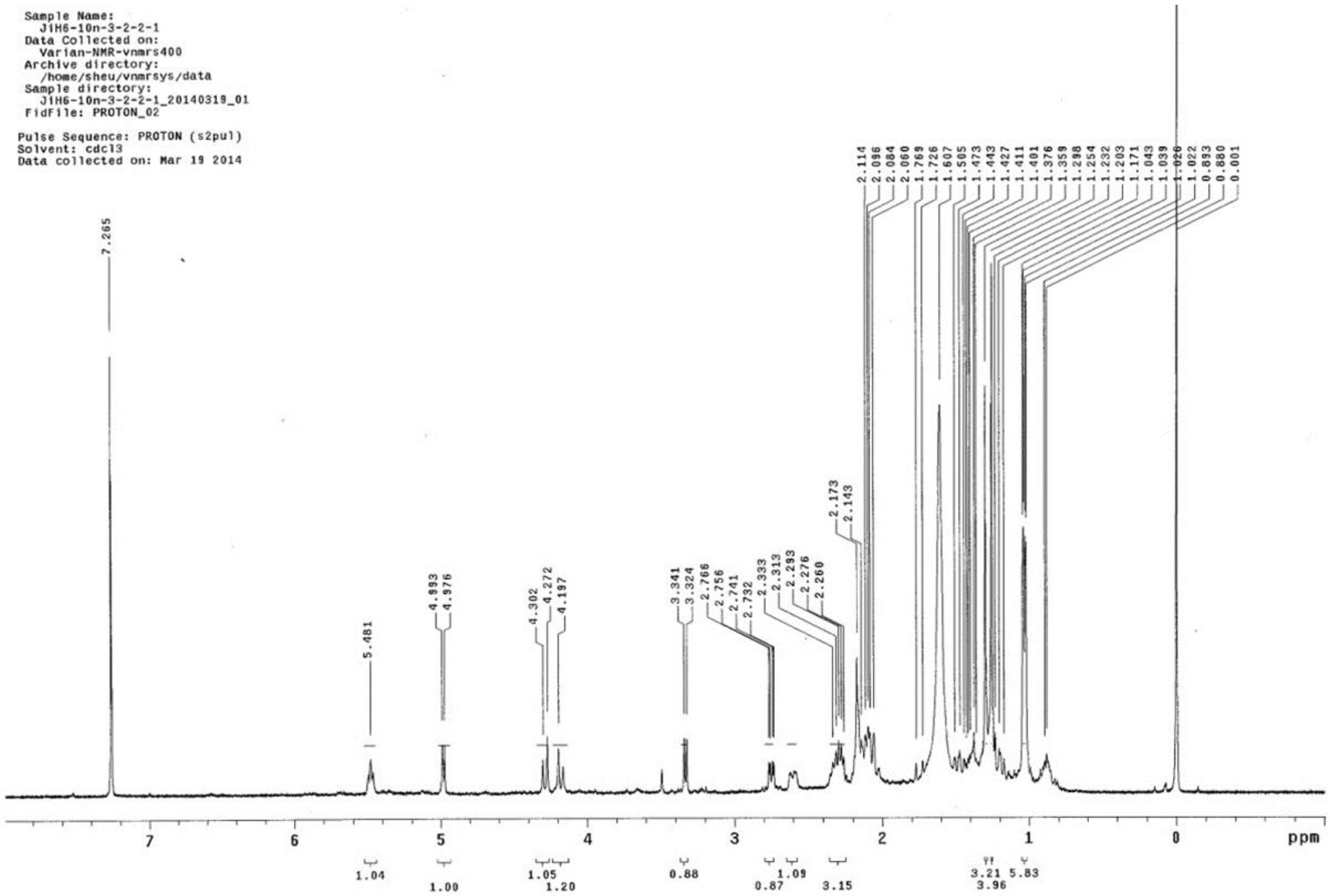

Figure S11. ^{1}H NMR spectrum of **4** in $CDCl_3$ at 400 MHz.

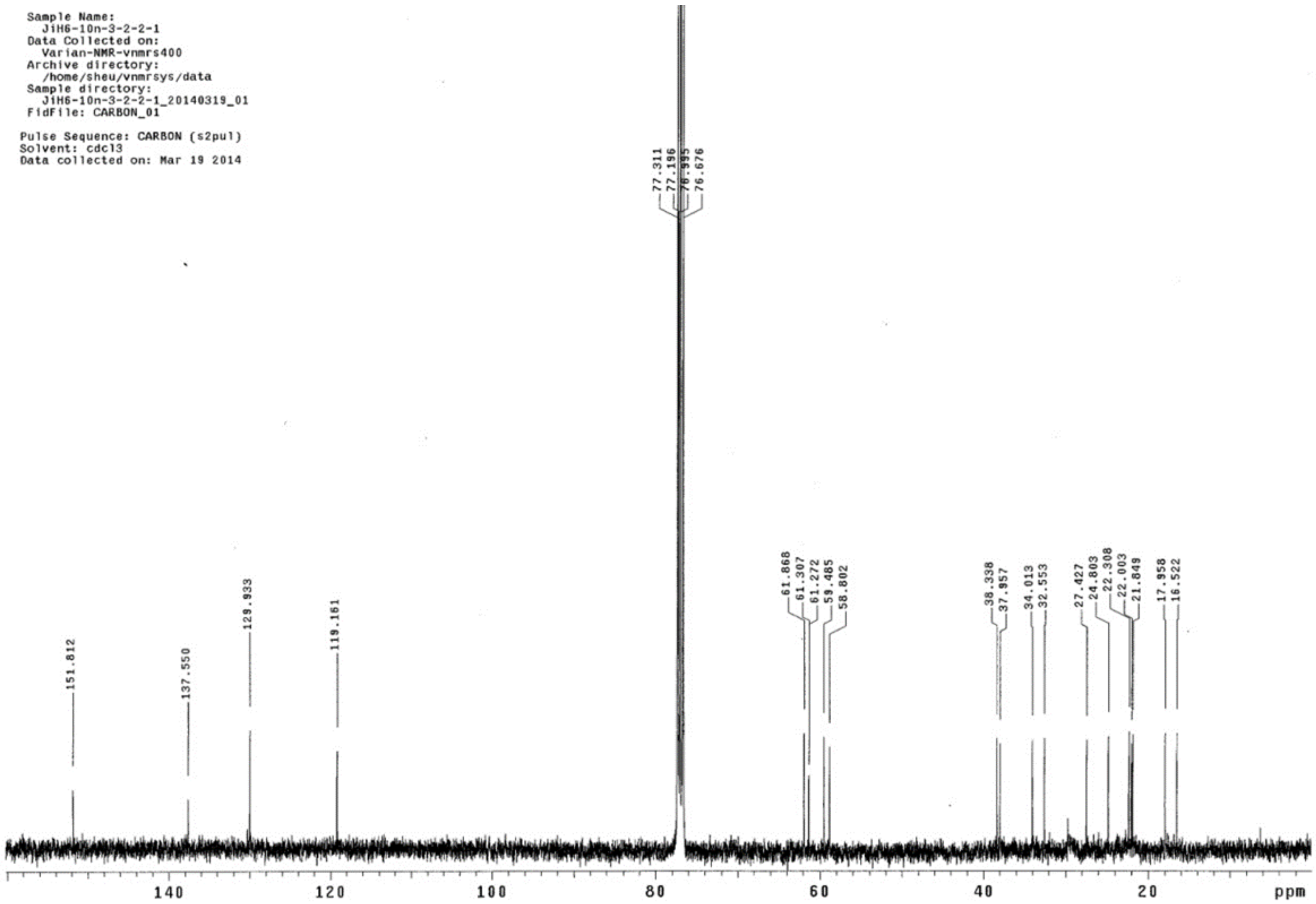

Figure S12. ^{13}C NMR spectrum of **4** in $CDCl_3$ at 100 MHz.

Mass Spectrum SmartFormula Report

Analysis Info

Analysis Name	D:\Data\a1\JIH614N641_000001.d	12/16/2013 11:30:59 AM
Method	broadband first signal	
Sample Name	JiH6-14n-6-4-1	Instrument: FT-MS solariX
Comment	ESI Positive	

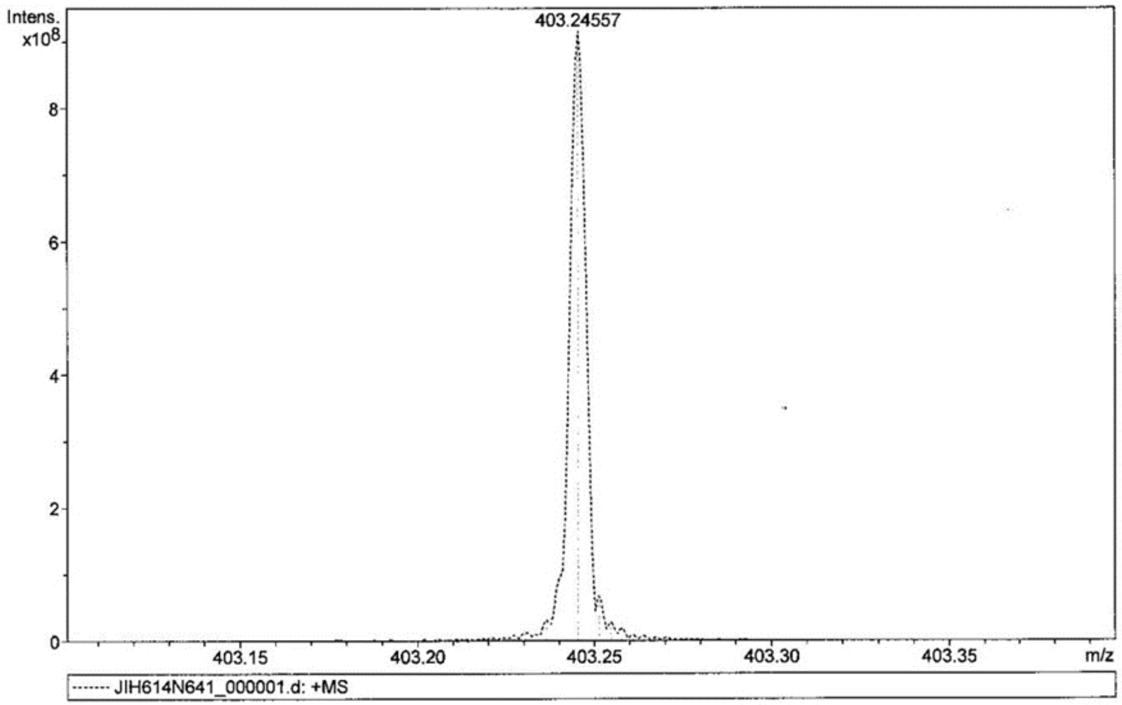

Meas. m/z	#	Formula	Score	m/z	err [mDa]	err [ppm]	mSigma	rdb	e⁻ Conf	N-Rule
403.24557	1	C 22 H 36 Na O 5	100.00	403.24550	-0.07	-0.18	38.1	4.5	even	ok

Figure S13. HRESIMS spectrum of **5**.

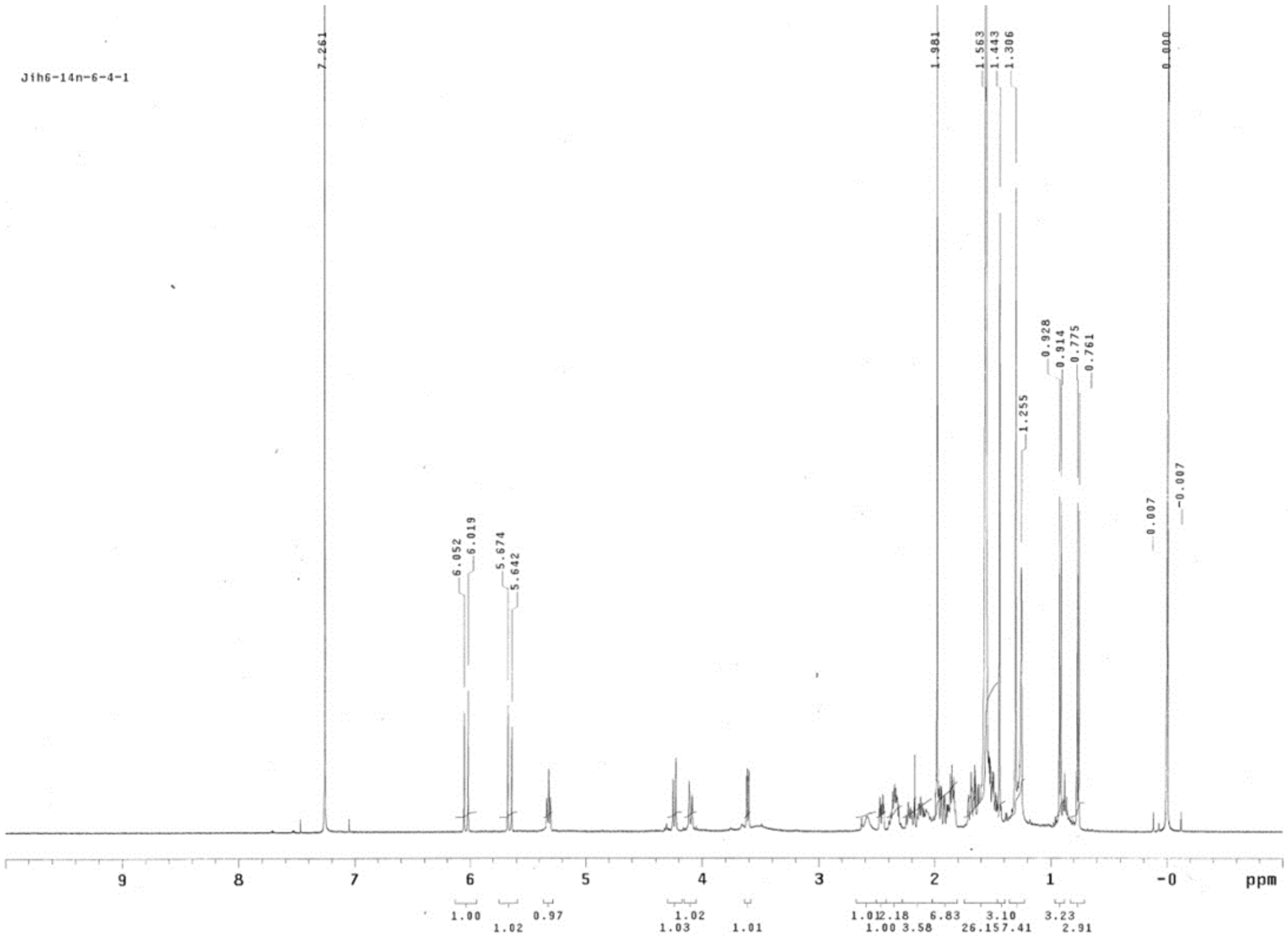

Figure S14. ^{1}H NMR spectrum of **5** in $CDCl_3$ at 500 MHz.

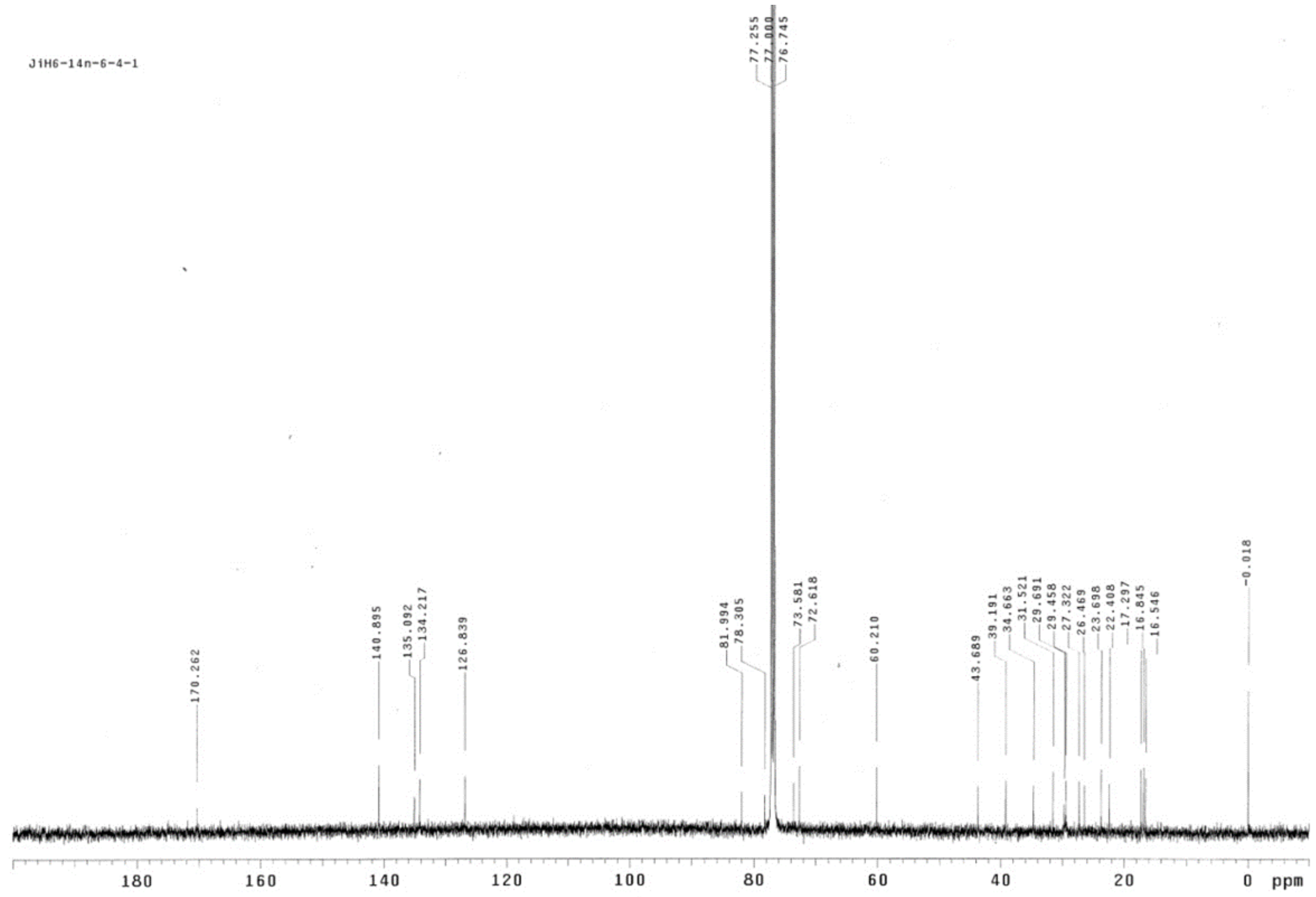

Figure S15. ^{13}C NMR spectrum of **5** in $CDCl_3$ at 125 MHz.
